# Enemies or Allies? Hormetic and Apparent Non-Dose-Dependent Effects of Natural Bioactive Antioxidants in the Treatment of Inflammation

**DOI:** 10.3390/ijms25031892

**Published:** 2024-02-04

**Authors:** Uxía Barreiro-Sisto, Sandra Fernández-Fariña, Ana M. González-Noya, Rosa Pedrido, Marcelino Maneiro

**Affiliations:** 1 Departamento de Química Inorgánica, Facultade de Ciencias, Campus Terra, Universidade de Santiago de Compostela, 27002 Lugo, Spain; uxiabarreiro.sisto@usc.es (U.B.-S.); sandra.fernandez.farina@usc.es (S.F.-F.); 2 Departamento de Química Inorgánica, Facultade de Química, Universidade de Santiago de Compostela, 15782 Santiago de Compostela, Spain; rosa.pedrido@usc.es

**Keywords:** inflammation, antioxidants, bioactive, hormetic effect, oxidative stress, non-dose-dependent, curcumin, resveratrol, ferulic acid, linoleic acid

## Abstract

This review aims to analyze the emerging number of studies on biological media that describe the unexpected effects of different natural bioactive antioxidants. Hormetic effects, with a biphasic response depending on the dose, or activities that are apparently non-dose-dependent, have been described for compounds such as resveratrol, curcumin, ferulic acid or linoleic acid, among others. The analysis of the reported studies confirms the incidence of these types of effects, which should be taken into account by researchers, discarding initial interpretations of imprecise methodologies or measurements. The incidence of these types of effects should enhance research into the different mechanisms of action, particularly those studied in the field of basic research, that will help us understand the causes of these unusual behaviors, depending on the dose, such as the inactivation of the signaling pathways of the immune defense system. Antioxidative and anti-inflammatory activities in biological media should be addressed in ways that go beyond a mere statistical approach. In this work, some of the research pathways that may explain the understanding of these activities are revised, paying special attention to the ability of the selected bioactive compounds (curcumin, resveratrol, ferulic acid and linoleic acid) to form metal complexes and the activity of these complexes in biological media.

## 1. Introduction

Different natural bioactive antioxidant compounds show beneficial effects for reducing oxidative stress [1,2,3,4,5,6,7,8]. Oxidative stress is generated by an imbalance between reactive oxygen species (ROS) and the body’s antioxidant system that can trigger inflammatory processes, as well as various pathologies [9,10,11,12,13]. ROS includes different molecular chemical species, such as hydrogen peroxide (H_2_O_2_), lipid hydroperoxide (LOOH), peroxynitrate (ONOO^−^), superoxide anion (O_2_^•−^) and other radicals such as hydroxyl (OH^•^), lipid radical (L^•^) or peroxyl radical (ROO^•^). Likewise, the antioxidant system that regulates the ROS level includes enzymes such as superoxide dismutase, catalase, glutathione peroxidase and other non-enzymatic species such as α-tocopherol, ascorbic acid and carotenes [14,15,16].

**Figure 1:**
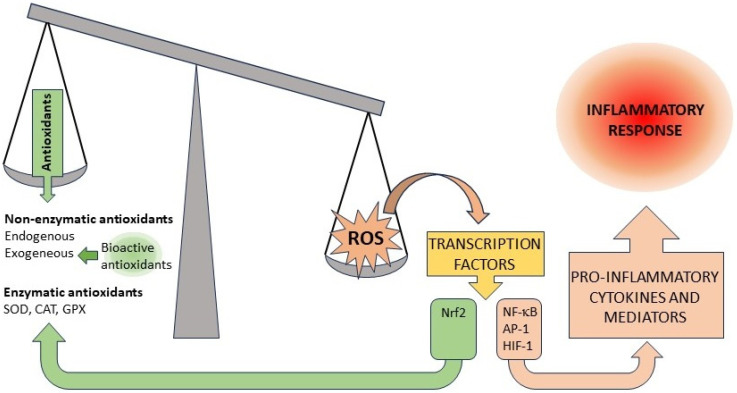
Representation of the imbalance between ROS and the body’s antioxidant system. An excess of ROS triggers the inflammatory response. SOD: superoxide dismutase; CAT: catalase; GPX: glutathione peroxidase; Nrf2: nuclear factor erythroid-derived 2; NF-κB: nuclear factor kappa-light-chain-enhancer of activated B cells; AP-1: activated protein 1 transcription factor; HIF-1: hypoxia-inducible factor 1.

The accumulation of ROS plays a key role in triggering the inflammatory process (Figure 1), because they can activate the receptors of immune cells and cause the release of inflammatory mediators [17,18]. In the context of this article, it is pertinent to review some basic notions about inflammation. It is important to keep in mind that it is part of the immune system’s defense response to a potential threat [19,20,21]. The inflammatory signal alerts the organism’s immune system to face this threat. The process can be triggered by multiple causes, because of an injury (trauma, radiation, cuts, burns, etc.), a pathogenic infection (virus, fungi, bacteria), an allergic reaction or a chemical irritant, among other reasons.

During the inflammatory response, different symptoms are generated in the organism, ranging from pain to loss of function of the affected tissue. These symptoms are related to the roles of different mediators, such as cytokines, which cause an increase in platelet reactivity or a decrease in natural anticoagulant systems. This defense mechanism is designed so that, once the threat is neutralized, it remits to acute inflammation and the clots formed are dissolved.

**Figure 2:**
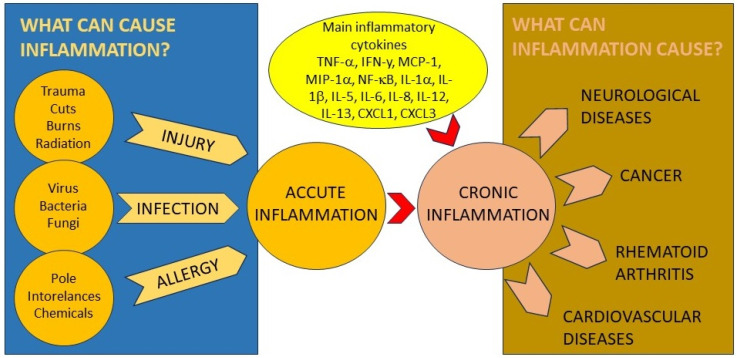
Some of the causes that produce acute inflammation, the main inflammatory cytokines involved in chronic inflammation and some of the diseases derived from this process. The term inflammation encompasses a complex process that can be triggered by multiple causes and can also produce multiple pathologies. TNF: tumor necrosis factor; IFN: interferon; MCP: monocyte chemoattractant protein; MIP: macrophage inflammatory protein; NF-κB: nuclear factor kappa-light-chain-enhancer of activated B cells; IL: interleukin; CXCL: chemokine (C-X-C motif) ligand.

Acute inflammation can, in some cases, lead to critical episodes for organisms. An example of this is the infection caused by SARS-CoV-2, which, in some individuals, can cause acute respiratory disease and severe pneumonia that can lead to death [22,23]. Regardless of the severity of these inflammation episodes, from the point of view of bioactive antioxidant compounds, the interest is focused on chronic inflammation processes, which occur when the inflammatory defense mechanism is maintained beyond the necessary time. Figure 2 collects some of the pro-inflammatory cytokines/pathway regulators, such as TNF-α, IFN-y or different interleukin (IL-1β, IL-5, IL-6, etc.), involved in the process of chronic inflammation [24]. Overexpression of these cytokines during inflammatory responses leads to an imbalance in their levels and may result in the development of different pathologies. The signs of chronic inflammation are not external; normally, they are subtler because the inflammatory response continues even though there is no injury or infection. This dysfunction facilitates the appearance of some chronic pathologies, such as cardiovascular diseases, neurological diseases, rheumatoid arthritis, cancer, etc. [25,26,27,28,29,30,31]. In this context, the use of antioxidant natural bioactive compounds to counteract the inflammatory process makes sense. Figure 2 shows some of the multiple causes that produce inflammation and also several of the different diseases that chronic inflammation can generate.

In this contribution, we aim to review the research in this field by focusing on the hormetic effects, with a biphasic response depending on the dose, and activities that are apparently non-dose-dependent [32,33,34,35,36,37]. In the first section, this type of behavior is defined and described in the context of the law of mass action. Then, the published research on bioactive antioxidants with these unexpected dose–response curves is analyzed, focusing on the most recent contribution. Hormetic effects have been found in many bioactive compounds, such as quercetin, catechin, gallic acid, rosmarinic acid, fucoxanthin, salvianolic acid, etc. [38]. To systematize this review and make it more approachable, we have focused the revision on four natural bioactive antioxidants: curcumin, resveratrol, ferulic acid and linoleic acid. The four compounds have different structural features, such as keto/enol tautomerism in curcumin or the aliphatic/aromatic nature of the two selected acids.

In the context of the benefits against inflammation, these natural bioactive compounds with hermetic behavior can generate a hermetic stress capable of activating the Nrf2 factor, a transcription factor that plays a fundamental role in regulating the cellular response to oxidative stress, protecting the cell from damage and preventing inflammation. The beneficial hermetic role of these bioactive compounds, in this case, is not so much due to their direct antioxidant action as to their pro-oxidative action to produce in the organism the so-called adaptative response to induced stress [39,40,41]. The discussion section of the review is devoted to considering the explanations that should lead to the understanding of the scientific bases of these unexpected behaviors. Among the complexity involved in the multiple processes that natural antioxidants can undergo in the body, this review also provides the viability of the coordination of these species to metal ions.

## 2. Dose–Response Curves, Hormesis and the Law of Mass Action

The law of mass action, formulated by Guldberg and Waage in 1867 [42], is a fundamental tool in chemical calculations. The simplicity of the statement, which indicates that the reaction rate is proportional to the product of the reactants concentrations raised to powers equal to their coefficients in the chemical equation, hides the potential to quantitatively predict the concentrations and rates of the different species in a dynamic equilibrium. Any chemical process follows this law, including those developed in biological media, and this allows us to calculate the concentrations of the reactants and products, as well as the different activity curves, as a function of the dose [43].

**Figure 3:**
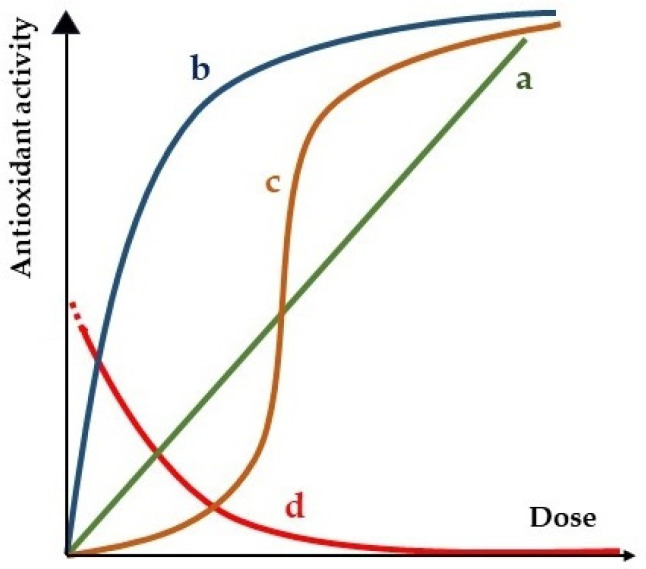
Dose–response curves for antioxidant compounds (adapted from Georgieva and Vassileva [58]). Curves a, b and c are representations of common non-threshold curves, that is, the effect appears at any concentration. In some cases, threshold curves are observed where the antioxidant activity arises at a certain minimal dose. (a) Linear response; (b) saturation curve; (c) sigmoid curve; (d) non-dose-dependent antioxidant activity response.

If we focus on the antioxidant activity of a compound, the simplest dose–response correlation would be the linear-no-threshold model, which is labelled as “a” in Figure 3, with an effect proportional to the administered dose. Although it is quite common for the effect to be observed only from certain concentrations, known as threshold curves, in this review we will not take this effect into account in the dose–response curves.

Linear or monotonic correlation occurs in the first interaction of a chemical species with a biochemical target molecule, but biological media are typically complex and give rise to nonlinear responses [44]. This is because the antioxidant compound can interact with multiple molecules and systems in the body, which can lead to complex effects [45,46,47,48,49,50,51]. In Figure 3, two representations of this type of nonlinear dose–response curve are labelled as “b” and “c”. Curve “b” corresponds to a saturation model and curve “c” to a sigmoidal behavior model. Both reach a maximum at a certain dose. This maximum represents the maximum antioxidant response that can be obtained with the compound. An explanation for this can be that the effect of the compound is limited by the number of receptors available.

In the literature, a number of antioxidant activities that are not apparently dependent on the dose have also been described [52,53,54,55,56,57,58,59,60,61,62,63]. The curve labelled “d” in Figure 3 represents a model for this type of behavior. These compounds are found to exhibit antioxidant-protective effects at low doses and even produce oxidative stress at high concentrations, which is an unexpected type of antioxidant activity. In the following sections of this review, the different explanations that can help us understand this type of dose–response curve will be discussed in more detail.

**Figure 4:**
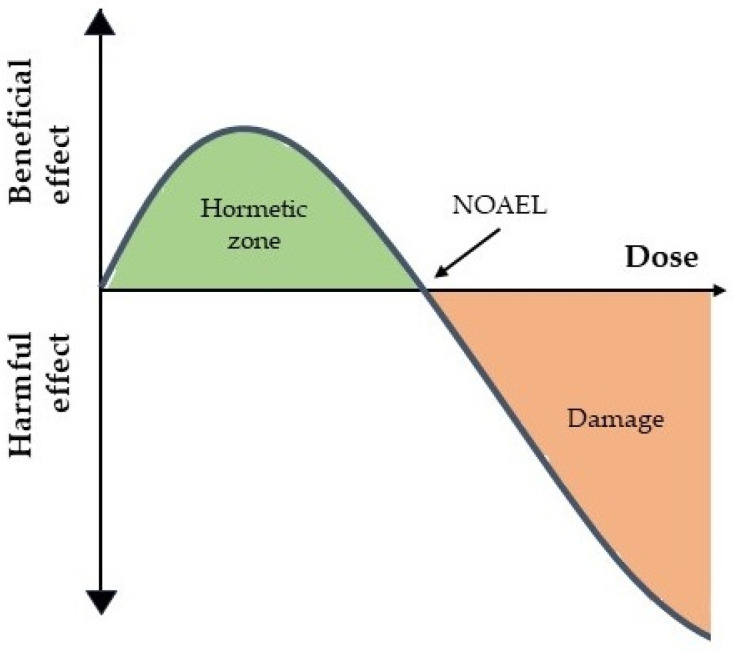
Biphasic hormetic curve. NOAEL is the No Observable Adverse Effect Level. Doses above this point cause damage to the organism. On the contrary, doses below the NOAEL generate a beneficial effect (adapted from Skaperda et al. [40]).

Among the biphasic responses produced by different drugs, with increasing interest, there is one known as the hormetic response [39,64,65,66,67,68,69,70,71,72,73]. If we focus on antioxidant activity, hormesis is characterized by a protective and beneficial effect at very low doses, while increasing the concentration of the antioxidant causes damage (Figure 4). The point from which the dose is harmful to the organism is called NOAEL (No Observable Adverse Effect Level).

This type of hormetic response was first reported in 1888 by Schulz [74] when he found that mercury chloride increased the fermentation capacity of yeast at very low concentrations. Therefore, it is a phenomenon that has been known for many years but has been marked by controversy for many decades [40,75,76]. This is fundamentally due to, on the one hand, the appropriation of some of the characteristics of the hormetic effect, which only appears at very low doses, by homeopathic philosophy. On the other hand, this positive response at low doses was the basis of the current defense in the environmental toxicology field, in many cases motivated by commercial interests in the use of some toxic compounds and the risk thresholds of toxic substances at low levels of exposure.

**Figure 5:**
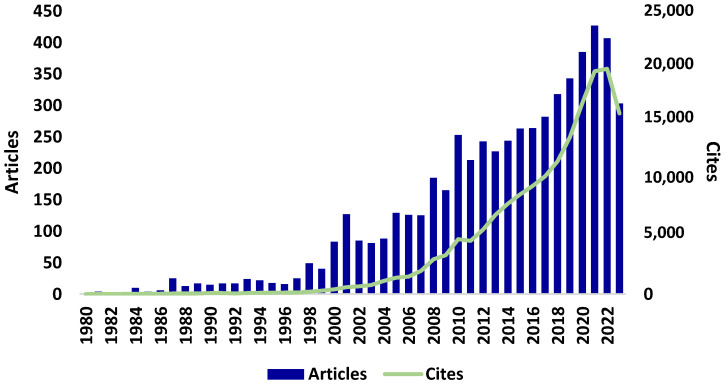
Number of citations and publications related to hormesis per year (1980–2023). The search was performed within the Web of Science with the keywords “hormesis” or “hormetic” (searched on 13 December 2023, 4:30 pm, Western European Standard Time).

In the last two decades, there has been a renewed interest in the scientific study of hormetic phenomena, with an accelerated increase in publications in this field (Figure 5). The overcoming of prejudices from previous decades has allowed the hormesis concept to be widely generalized. Therefore, researchers now have more freedom to present their results with unexpected dose responses, or that imply explanations that, apparently, were not in line with the conventional interpretation of the law of mass action.

It is likely that, when the mainstream questioned these dose–response curves, many researchers were reluctant to publish results in this direction. What is evident is that the number of citations regarding this phenomenon was residual for many years, in contrast to the current situation.

## 3. Unexpected Dose–Response Curves in the Antioxidant and Anti-Inflammatory Activity of Natural Bioactive Compounds

The beneficial effects of natural bioactive compounds on inflammatory processes have been extensively researched. In this sense, the literature offers numerous examples that address the results of these investigations [1,2,8,77,78,79,80,81]. In the present review, we will focus on those in which unexpected dose–response curves are described. A significant number of these examples correspond to plant extracts [52,53,55,56,57,59,60,62,63], with a composition not accurately defined, so they will not be addressed in this section. To systematize the study in this part of the revision, four natural bioactive compounds have been selected: curcumin, resveratrol, ferulic acid and linoleic acid.

In this work, the objective is not a detailed review of each of these natural bioactive compounds, but rather to show that these unexpected dose–response curves are more common now than they were in the literature of a few years ago, without being restricted to very limited compounds.

### 3.1. Curcumin

**Scheme 1:**
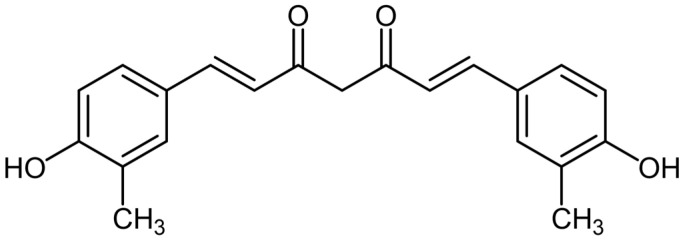
Structure of curcumin: (1*E*,6*E*)-1,7-bis (4-hydroxy-3-methoxyphenyl)-1,6-heptadiene-3,5-dione.

Curcumin is extracted from turmeric, a species obtained from the root of *Curcuma longa* which has been used since ancient times for various applications [82]. It is a diarylheptanoid (Scheme 1) that exhibits tautomerism between the keto form (predominant in polar solvents) and the enol form, which is stabilized by the formation of intramolecular hydrogen bonds when dissolved in polar solvents. It is a natural bioactive compound with antioxidant and anti-inflammatory activity [83,84].

The hormetic effects of curcumin are reported in multiple examples in the literature. Several recent reviews that analyze in detail the conditions of these experiments, results, and proposed mechanisms to understand these behaviors are available to researchers [85,86,87,88,89].

Studies report different types of hormetic effects attributable to curcumin. In this way, the ability to affect the cell proliferation of some cell lines has been described, with stimulation for low doses of curcumin and suppression of stimulation when the dose is increased [90,91,92,93]. Kim et al. [90] found that low doses (0.1–0.5 μM) of curcumin increased neural progenitor cell proliferation (NPC), whereas high doses (>10 μM) became cytotoxic. In the case of the research by Santel et al. [91], a concentration of more than 50 μM was sufficient to suppress cell proliferation via inhibition of glyoxalase 1 (Glo1). Lee et al. [92] reported a regulatory effect of the AMPK-COX-2 signaling pathway on HT-29 colon cancer cells at curcumin concentrations of 50 μM and above. Son et al. [93] observed an increase in NPC proliferation at low doses of curcumin (0.1–1 μM) via a mitogen-activated protein kinase signaling pathway, whereas high doses (>10 μM) caused a decrease in cell proliferation.

In the context of the anti-inflammatory activity of curcumin, Rattan et al. [94,95,96] have described its ability to promote the repair of damaged tissues in wounds and their healing at low concentrations (0.25–5 μM). Conversely, at higher concentrations (10 μM), curcumin delays the wound healing process. The authors described a biphasic effect of curcumin on the Na,K-ATPase sodium pump. They found that this natural bioactive compound stimulates proteasome activity, enhances heat shock protein induction after a heat shock response, and stimulates sodium pump activity at low doses.

More recently, Tianzheng et al. [97] found that curcumin (dose 5 μM) can regulate ROS hormesis promoting mitochondrial fusion/elongation, biogenesis and improved function in C57BL/6J model mice through an NADPH oxidase-dependent redox signaling pathway. Stepien et al. [98] have proposed that curcumin is able to delay the aging of a wild-type strain of yeast, probably by a hormetic process.

Some research studies of curcumin report behaviors that are not dose-dependent, although these results are not referred to as products of hormesis [99,100,101,102]. For instance, Hang et al. [99] reported antioxidant properties of curcuminoids with a non-dose-dependent effect. Gomes et al. [100] also reported non-dose-dependent plasma curcumin levels in a study where low doses of *Curcuma longa* improved the antioxidant capacity of subjects, whereas higher doses had a negative effect on antioxidant capacity. However, this pilot study was limited to a small number of nine healthy subjects. Therefore, these non-dose-dependent behaviors would have to be taken with caution. When studies are conducted with individuals, the bioavailability of curcumin can be affected by various factors depending on the assimilation processes after oral ingestion by individuals [101]. This makes it difficult to draw conclusions about the causes leading to the activities that appear to be independent of the dose.

### 3.2. Resveratrol

**Scheme 2:**
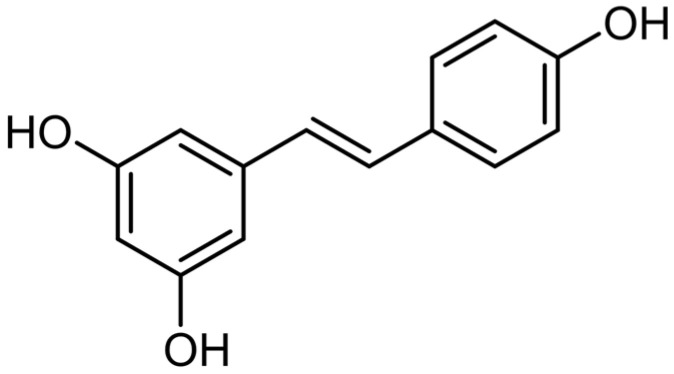
Structure of resveratrol: 3,5,4′-trihydroxy-*trans*-stilbene.

Resveratrol is a hydroxylated derivative of stilbene, produced by plants such as grapes, berries and peanuts [103], that is used as a phytoalexin in response to pathogen infections [104]. It shows geometric isomerism with cis/trans isomers. Scheme 2 shows the structure of the trans isomer. The antioxidant properties of resveratrol have been studied extensively, as the high concentration in fresh grape skin (50–100 mg per gram) also produces important levels of this active ingredient in red wine. This has led to interest from those involved in the production and commercialization of red wine in establishing the potential beneficial effect of resveratrol [105].

The hormetic effects of resveratrol have already been discussed in the literature as it exhibits a dose-dependent biphasic effect [41,106,107,108,109,110,111,112,113]. Thus, resveratrol can induce opposite effects in different doses. Plauth et al. [41] reported the increase in the cell viability at low concentrations of resveratrol (less than 50 μM) in a set of representative cell models under oxidative stress, while higher concentrations decrease viability. In this study, they reported that the hormetic effects of oxidative products derived from resveratrol are driven by activation of Nrf2.

Dai et al. [113] reported the stimulation of cell proliferation and osteogenic differentiation of bone marrow stem cells via ER-dependent ERK1/2 activation, enhancing resilience to inflammatory stress, at low concentrations of resveratrol, which became inhibitory at higher concentrations of 50 μM. Caldarelli [114] also found that resveratrol can enhance differentiation, again with a biphasic hormetic effect, of human bone marrow mesenchymal stromal cells but a lower concentration (0.5–1.0 μM).

More recently, Calabrese [115] reviewed the occurrence of hormetic dose responses induced by resveratrol in the repairing of damaged endothelial cells due to traumatic injury. Different studies support the capacity of resveratrol to enhance tissue repair at low concentrations in a hormetic-type response [116,117]. Gu et al. [116] reported an acceleration in the reendothelization rate at low doses of resveratrol (10 mg/kg) in a rat model of an injured aorta, whereas a large dose (50 mg/kg) suppressed the beneficial effect. Xia et al. [117] found that resveratrol reduces endothelial progenitor cell senescence through protein kinase B-dependent mechanisms. In this case, the maximal inhibitory effect is achieved at 50 μM of resveratrol, but an increase in senescence was observed at 100 μM.

In the literature, there are other studies in which the activity of resveratrol does not depend on the dose, but those are not classified as hormetic responses [118,119,120]. However, as was the case with the examples of this type using curcumin, these studies were performed with a reduced number of subjects, so they must be taken with some caution.

Another factor to consider is whether or not the studies have been conducted in the correct dose range to detect the dose-related effect, since, if the studies only cover a narrow range of doses, the dose-dependent effect may be missed.

### 3.3. Ferulic Acid

**Scheme 3:**
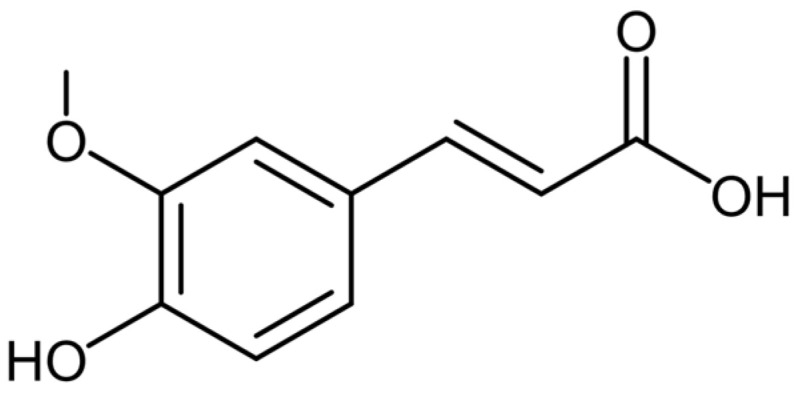
Structure of ferulic acid: (2*E*)-3-(4-hydroxy-3-methoxyphenyl)prop-2-enoic acid.

Ferulic acid (Scheme 3) is a phenolic acid of low toxicity commonly found in vegetables, fruits and maize bran, and is always found covalently conjugated though ester-linkage with saccharides, glycoproteins and other biomolecules [121]. Ferulic acid presents numerous biological properties and has been employed for the treatment of several free radical-induced disorders due to its antioxidant properties [122].

Regarding the hormetic role of the ferulic acid, it is known to be very important in maintaining the homeostasis of the organisms. Due to this fact, different detailed studies on the hormetic effects of this bioactive compound have been reported [38]. Ferulic acid presents anti-inflammatory and antioxidant properties and was selected for use in improving neuroprotection. Thus, at high doses (>100 μM), it was shown to be inhibitory to angiogenic endpoints, whereas lower doses (10 μM) revealed responses related to cell viability (MTT assay), cell migration and tube formation [123]. Therefore, it was demonstrated by Moghadam et al. [124] that ferulic acid shows neuroprotective and proliferative properties at small concentrations (50 μg/mL) through stabilization and degradation of the tumor suppressor protein p53. This is attributed to an increase in the expression rate of sirtuin proteins SIRT1 and SIRT7, and the negative regulator MDM2, but a down-regulation in USP7. However, at high concentrations (250–800 μg/mL), it induces neuronal difference and neurite growth.

### 3.4. Linoleic Acid

**Scheme 4:**
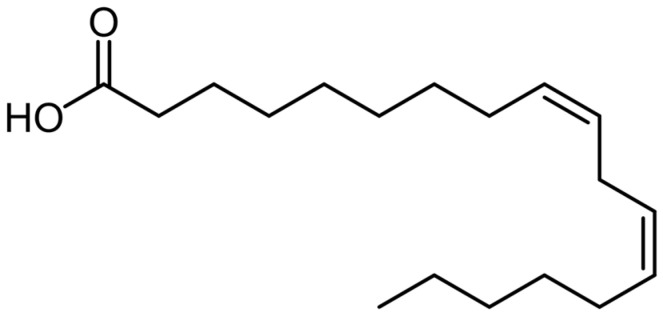
Structure of linoleic acid: (9*Z*,12*Z*)-Octadeca-9,12-dienoic acid.

Linoleic acid (Scheme 4) can be found in vegetable oils and is the most important fatty acid. It is essential for human growth since it plays a physiological role due to its antioxidant properties; for example, in preserving the water permeability barrier of the skin, in has a protective effect against cancer and atherosclerosis, as well as in the production of proinflammatory substances involved in the regulation of inflammatory responses [125,126,127].

Different studies have demonstrated that linoleic acid also induces a biphasic response, depending on the dose. Fang et al. [128] explored the influence of different fatty acids, including linoleic acid, on the lifespan of *Caenorhabditis elegans*, a common model for exploring antioxidant and aging properties due to its similarity with humans in its physiological processes and regulatory mechanisms. In this study, it was found that low concentrations of linoleic acid increased lifespan while high concentrations decreased it, with the most effective antiaging concentration being 0.5 mg/plate, increasing the lifespan by 8.47% due to the activation of the caloric restriction mechanism, which is a type of stress relevant to the hormetic mechanism.

Recently, Bergamo et al. [129] demonstrated that small concentrations (600 mg/kg) of conjugated linoleic acid (CLA) increase the levels of NADPH oxidases (NOX), one of the major sources of cellular ROS, thus enhancing hepatic redox status. However, a high concentration (3000 mg/kg) of CLA is associated with the modification of lipid homeostasis or mitochondrial functions. The authors attributed the observed hermetic effects produced by CLA on the dose-dependent fluctuation on the Nfr2/PGC-1α pathway.

## 4. Discussing Unexpected Dose–Response Curves in Natural Bioactive Compounds

In the previous sections, we have emphasized the occurrence of the biphasic response, as a function of the dose, of different natural bioactive compounds. The hormetic response is widespread and, without a doubt, it is an expected behavior that must be taken into account. This hormetic response can be fully adjusted to the law of mass action. Hormetic curves do not occur in first-order reactions between two isolated chemical species (which we could classify as deterministic), but rather nonlinear responses appear in more complex or stochastic biological media, characterized by the lack of predictability of a high set of cellular responses [44,48,49].

Scientists use statistical tools to analyze stochastic processes. Thanks to statistics, it is possible to calculate the probability distribution (the range of probable results of a given experiment) or determine the probability of a specific event [130]. Part of the advancement of science in the context of the in vitro and in vivo trials of different active compounds is based on these statistical calculations, even if the underlying biochemical mechanisms are not fully understood.

Different authors have tried to interpret the hormetic effects of different compounds. In some cases, well-documented opposing effects on cell proliferation and mitochondrial redox metabolism can explain biphasic dose–response curves [44,45,46,47,48,49,50,51]. This has allowed modeling of some biphasic dose–response curves, which is possible when the conditions of a limited number of biochemical reactions that generate opposing effects in individuals are known [46,47,48,49,50,51].

In the case of curves that are apparently non-dose-dependent, their modeling is complicated in a way that is consistent with the law of mass action, and it has already been commented that, at least in some cases, the dose range has not been correctly selected to detect the dose-dependent effect [48,54]. This can happen in those cases of hyper-susceptibility reactions, which can occur, for example, with some immunological markers, and where the adverse effect reaches a maximum at doses lower than those studied by the authors. In these cases, curves that apparently are not dose-dependent can mask a hormetic effect that could be detected at lower concentrations. It is also possible that these responses hide other unforeseen effects, such as reactions that are related to a cumulative dose or saturation of the absorption of the compound by the individual. Another issue to consider is, as already indicated, that some of these studies have been conducted with a limited number of subjects, which can reduce the statistical validity of the results [100,101,118,119,120].

Returning to the hormetic effect, its interpretation requires knowledge of the different reactions that compounds can give rise to in the body. Other authors have reviewed the interactions of different natural bioactive compounds, such as those analyzed in this review (curcumin, resveratrol, ferulic acid or linoleic acid), with transcription factors, such as the inhibition of NF-κB activity or the activation of ERK1/2, CREB or the Nrf2 pathway [39,40,41,66,67,68,70]. In contrast, in this work, we will highlight another type of process that can produce these natural bioactive compounds, such as the formation of complexes with different metals.

**Figure 6:**
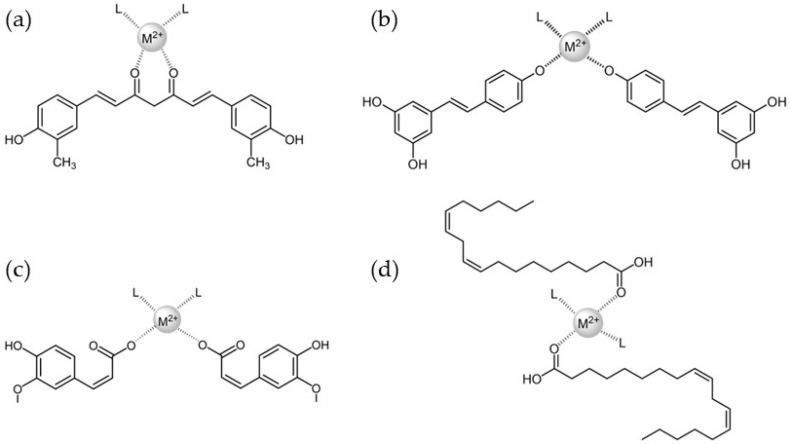
Representation of the coordination of different natural bioactive compounds to divalent metal ions: (**a**) metal complex with curcumin acting as a chelate ligand; (**b**) metal complex with two resveratrol moieties; (**c**) metal complex incorporating two ferulic acid molecules; (**d**) metal complex with linoleic acid as a monodentate ligand.

Curcumin, resveratrol, ferulic acid or linoleic acid have in common that they can act as ligands to coordinate metal ions in the body; they all have atoms that can act as charge donors towards the free orbitals of different metal ions. Figure 6 shows some metal complexes that can be formed with these natural bioactive compounds.

In fact, this type of coordination compound is well documented in the literature. Table 1 shows a significant number of metal complexes (with Co^2+^, Ni^2+^, Cu^2+^, Zn^2+^, Mn^2+^, Mg^2+^, VO^2+^, Fe^2+^, Fe^3+^, Ga^3+^, Pt^2+^, Ru^2+^, Na^+^) where curcumin acts as a ligand [131,132,133,134,135,136,137,138,139,140,141,142,143,144,145,146,147,148,149,150,151,152], limited only to the period 2018–2023, and for those cases where the antioxidant or anti-inflammatory activity of the metal complexes has been studied in parallel. In general, metal compounds show better antioxidant/anti-inflammatory behavior in published studies than free curcumin.

The hydroxyl group attached to the carbon chain plays a key role in the antioxidant properties of curcumin. Although the antioxidative mechanism of this bioactive compound is not fully understood, it is generally accepted that it involves the direct transfer of a hydrogen atom from this hydroxyl group of the antioxidant to the free radical [139,145,153,154]. This hydrogen atom transfer allows curcumin to modulate the inflammatory response by inhibiting the activity of inflammatory cytokines IL-1, IL-2, IL-6, IL-8 and IL-12, and by down-regulating the activity of other molecular targets involved in inflammation [138,155].

The enhancement of the antioxidant and anti-inflammatory properties of curcumin upon complexation with a variety of metals has been explained in the literature based on various factors. Halevas et al. [139] pointed out how complexation may increase the reducibility of the natural polyphenol, but complexation may also modulate its stability and solubility [138,139]. Other authors [133,146] highlighted the SOD-like activities of the metal–curcumin complexes. Curcumin coordination compounds with Cu(II) or Zn(II) have shown an increase of the activities of antioxidant enzymes (CAT, SOD or GSH). In these cases, the mechanistic studies have shown the potential of these metal complexes to down-regulate inflammatory markers such as the nuclear factor NF-κB or the cytokines TGF-β and IL-8 [146].

The capacity of the curcumin complexes to act as metal carriers, therefore increasing their bioavailability, may also explain the anti-inflammatory effect for alkaline (Na^+^) or alkaline earth (Ca^2+^) metal complexes [138,148].

Without carrying out an analysis as exhaustive as in the case of curcumin, research into resveratrol, ferulic acid or linoleic acid coordinating with different metal ions can also be found. In the case of resveratrol, the literature reports [156,157,158,159,160] different coordination complexes with Mn^2+^, Cu^2+^, Al^3+^, Zn^2+^, Fe^2+^, Ca^2+^ or Mg^2+^. For ferulic acid, metal coordination complexes with Co^2+^, Ni^2+^, Cu^2+^, Zn^2+^, Mn^2+^, Mg^2+^, Fe^2+^, Fe^3+^ and Na^+^ are found [161,162,163,164,165,166,167,168,169,170]. For linoleic acid, metal complexes with Fe^2+^, Fe^3+^, Cu^2+^ and Pt^2+^ are described [171,172,173]. In general, metal complexes again show better antioxidant/anti-inflammatory behavior than the free ligands.

The quantity and variety of stable metal complexes that these natural bioactive compounds can form adds another factor to consider when interpreting the different responses that organisms can generate to these bioactive compounds. The ability of these bioactive compounds to stabilize metal complexes can allow them to act as metal ion chelators, but research also points to an increase in the antioxidant capacity of metal complexes compared to free bioactive compounds.

## 5. Future Prospects

Based on all of the above, it seems that the beneficial effects of natural bioactive compounds, such as curcumin, resveratrol, ferulic acid or linoleic acid, depend on the dose. As Paracelsus already advanced in 1538, only the dose makes the poison. In this review, a significant number of publications that report the beneficial effects of these natural bioactive compounds at low concentrations have been described, in contrast to the damage that they may cause at higher doses.

There is still a great lack of knowledge about the mechanisms of action that govern the hormetic effects of some natural bioactive compounds. The conclusions are based, in many cases, on the results of the benefit/harm balance that statistical studies performed on a number of populations over a certain period of time have yielded. It is necessary to study the biochemical mechanisms involved in this type of behavior to unravel and interpret the responses they generate in living organisms. Basic research in this sense must accompany statistically analyzed data. Only the ultimate understanding of the molecular mechanisms involved will be able to both avoid the possible adverse effects of these compounds and take advantage of the potential benefits that they can generate. Antioxidative and anti-inflammatory activities in biological media should be addressed beyond a mere statistical approach.

The ability of many of these natural bioactive compounds to bind to metals and form metal complexes should be analyzed in more detail to determine how it interferes with the bioavailability in the organism of the natural bioactive compounds, their metal detoxification capacity or the possible activities in the biological medium of the formed metal complexes.

In the context of inflammation, it is essential to elucidate the consequences that can be produced by the action of compounds with hormetic activity, with pro-oxidative capacity, to activate the antioxidant defense system. Inflammation is a complex process with multiple causes and responses, as discussed in the introductory chapter and illustrated in Figure 2. Therefore, it is important to rule out the possibility that the prolonged action of some compounds with hormetic responses, such as very low concentrations of environmental agents, such as toxins, metals, suspended particles, etc., may cause a harmful long-term effect on the functioning of the antioxidant system and be responsible for some manifestations of chronic inflammation.

## Figures and Tables

**Table 1:** Reported metal complexes in the period 2018–2023 incorporating curcumin as a ligand with biological activity of significance in the context of this review.

Metal Ion/s	Biological Activity	Dose	Ref.
Co^2+^, Ni^2+^, Cu^2+^, Zn^2+^	Enhance antioxidant and anti-inflammatory activity after complex formation. Albino rats were used for in vivo studies.	10^−4^ mM for radical scavenger activity; 0.1 mL of a 1% *w*/*v* solution for in vivo studies.	[131]
Cu^2+^, Co^2+^, Ni^2+^, Zn^2+^	Complexes better antioxidant activity than free curcumin.	5–20 μM	[132]
Cu^2+^, Zn^2+^	Complexes better antioxidant activity than free curcumin. Complexes exert neuroprotective effects.	25 µM	[133]
Mn^2+^, Mg^2+^, Cu^2+^, Zn^2+^	Antioxidant activity of the complexes inhibiting lipid peroxidation in the rat tissue model.	0.1–10 μM	[134]
VO^2+^	Complexes better antioxidant activity than free curcumin.	1–25 μM	[135]
Zn^2+^, Cu^2+^, Fe^3+^	Complexes better antioxidant activity than free curcumin.	100 μg/mL	[136]
Cu^2+^	Complex more potent antioxidant agents than curcumin. Activity influenced by the metal–ligand molar ratio.	0.3–0.9 nM	[137]
Fe^2+^, Zn^2+^, Ca^2+^	Complexes more potent antioxidant agents than free curcumin. Anti-inflammatory activity shown in Wistar albino rats.	200 ppm, 3 times daily, 3–21 days.	[138]
Cu^2+^	Complex more potent antioxidant agents than free curcumin.	2–20 μM	[139]
Mg^2+^, Ca^2+^	No difference in antioxidant activity between curcumin and metal complexes.	19–21 μM	[140]
Cu^2+^, Zn^2+^, Mg^2+^, Al^3+^, Fe^3+^	Complexes better antioxidant activity than free curcumin.	Complexes were incorporated to form hybrid composites.	[141]
Cu^2+^, Mg^2+^	Complexes better antioxidant activity than free curcumin.	10–11 μM	[142]
Cu^2+^, Co^2+^, Ni^2+^, Zn^2+^	Complexes better antioxidant activity than free curcumin.	250–1000 μg/mL	[143]
Ga^3+^	Complexes antioxidant activity close to free curcumin.	20–32 μM	[144]
Pt^2+^	No difference in antioxidant activity between curcumin and metal complexes.	10–50 μM	[145]
Zn^2+^	Complexes better antioxidant activity than free curcumin.	12–48 mg/kg Sprague–Dawley rats.	[146]
Zn^2+^	Complexes better antioxidant activity than free curcumin.	6.25–50 μM	[147]
Na^+^	Complexes better antioxidant and analgesic activities than free curcumin. Sprague–Dawley rats used for anti-ulcer studies.	0.1–15 µg/mL for antioxidant studies; 100 mg/kg body weight for in vivo studies.	[148]
Cu^2+^	Complexes better antioxidant activity than free curcumin.	1.24 μM	[149]
Ru^2+^	Complexes better antioxidant activity than free curcumin.	20–100 µg/mL	[150]
Mn^2+^, Co^2+^, Ni^2+^, Cu^2+^, Zn^2+^	Free curcumin better antioxidant activity than metal complexes.	6.25–100 μg/mL	[151]
Zn^2+^, Cu^2+^	Complexes exhibit antioxidant activity.	0.125–1.0 mg/mL	[152]

## Data Availability

Not applicable.
